# Multivariate Analysis of Water Quality and Benthic Macrophyte Communities in Florida Bay, USA Reveals Hurricane Effects and Susceptibility to Seagrass Die-Off

**DOI:** 10.3389/fpls.2018.00630

**Published:** 2018-05-08

**Authors:** Amanda M. Cole, Michael J. Durako, Margaret O. Hall

**Affiliations:** ^1^Department of Biology and Marine Biology, Center for Marine Science, The University of North Carolina Wilmington, Wilmington, NC, United States; ^2^Florida Fish and Wildlife Research Institute, Florida Fish and Wildlife Conservation Commission, St. Petersburg, FL, United States

**Keywords:** seagrasses, macroalgae, water quality, Florida Bay, multivariate analyses, hurricanes, die-off

## Abstract

Seagrass communities, dominated by *Thalassia testudinum*, form the principal benthic ecosystem within Florida Bay, Florida USA. The bay has had several large-scale seagrass die-offs in recent decades associated with drought and hypersaline conditions. In addition, three category-5 hurricanes passed in close proximity to the bay during the fall of 2005. This study investigated temporal and spatial trends in macrophyte abundance and water quality from 2006 to 2013 at 15 permanent transect sites, which were co-located with long-term water quality stations. Relationships, by year and by transect location (basin), between antecedent water quality (mean, minimum and maximum for a 6-month period) and benthic macrophyte communities were examined using multivariate analyses. Total phosphorus, salinity, pH, turbidity, dissolved inorganic nitrogen (DIN), DIN to phosphate ratio (DIN:${\text{PO}}_{4}^{-3}$), chlorophyll *a*, and dissolved oxygen correlated with temporal and spatial variations in the macrophyte communities. Temporal analysis (MDS and LINKTREE) indicated that the fall 2005 hurricanes affected both water quality and macrophyte communities for approximately a 2-year period. Spatial analysis revealed that five basins, which subsequently exhibited a major seagrass die-off during summer 2015, significantly differed from the other ten basins in macrophyte community structure and water quality more than 2 years before this die-off event. High total phosphorus, high pH, low DIN, and low DIN:${\text{PO}}_{4}^{-3}$, in combination with deep sediments and high seagrass cover were characteristic of sites that subsequently exhibited severe die-off. Our results indicate basins with more mixed seagrass communities and higher macroalgae abundance are less susceptible to die-off, which is consistent with the management goals of promoting more heterogeneous benthic macrophyte communities.

## Introduction

Seagrasses form the dominant benthic ecosystem in Florida Bay; they are also the dominant physical structure in the bay (Hall et al., 2007; Herbert et al., 2011). The most common seagrass species in Florida Bay are *Thalassia testudinum, Halodule wrightii*, and *Syringodium filiforme* (Hall et al., 1999, 2007). *Thalassia testudinum*, by far the most abundant seagrass, is a slow-growing climax-successional marine species that shows resilience and slow responses to changing conditions, while *H. wrightii* and *S. filiforme* are less resilient to change and more temporally dynamic (Williams, 1990; Whitfield et al., 2004; Hammerstrom et al., 2007). Historic changes in water usage in the Everglades have caused a 60% decline in freshwater inflow into the Florida Bay area (Herbert et al., 2011). These historic changes have caused an increase in salinity in Florida Bay, with increased occurrences of hypersalinity and reduced salinity variability (Herbert et al., 2011). The decrease in freshwater inflow has also caused changes in benthic community structure (Brewster-Wingard and Ishman, 1999; Halley and Roulier, 1999). Specifically, the reduction in freshwater runoff and resulting high salinity has led to “marinization” and increased homogeneity in the vegetation in the system, causing *T. testudinum* to become more widespread and abundant as other seagrasses have declined in abundance (Zieman, 1982; Fourqurean and Robblee, 1999).

Seagrasses serve as “coastal canaries,” which indicate problems due to environmental stressors or disturbances. These stressors include sediment and nutrient runoff, physical disturbances, invasive species, commercial fishing practices, aquaculture, disease, overgrazing, algal blooms, and global climate change, which have led to large-scale losses of seagrasses worldwide (Orth et al., 2006). A large-scale seagrass die-off occurred in Florida Bay from 1987 to 1991, with complete mortality of more than 4,000 ha of *T. testudinum* and additional negative effects in another 23,000 ha (Robblee et al., 1991). High salinity, elevated water temperatures, and water column stratification leading to bottom water anoxia, hypoxic stress, and sulfide toxicity are the main proposed mechanisms for initiating this die-off (Koch et al., 2007; Hall et al., 2016). The combination of these potential causative factors represents an example of the complex stresses brought on by climate and anthropogenic changes, which can cause a decrease in growth, distribution, and abundance of seagrass species (McKenzie et al., 2007). The ecological effects of this mass seagrass die-off and subsequent cascading disturbances (Butler et al., 1995) began events that led to the creation of the Comprehensive Everglades Restoration Program (CERP, 2000). A major objective of CERP is to alter the volume, distribution, and timing of freshwater inflow into Florida Bay. The ecological goal is to return the bay to more historic conditions, which includes a heterogeneous seagrass community that may be more resilient to disturbance than a community made up primarily of *T. testudinum*. In hypothetical restoration scenarios, only when mean salinity values are drastically lowered are *H. wrightii* and *Ruppia maritima* able to outcompete *T. testudinum* to form a more mixed community (Fourqurean et al., 2003; Lirman and Cropper, 2003).

**Figure 1:**
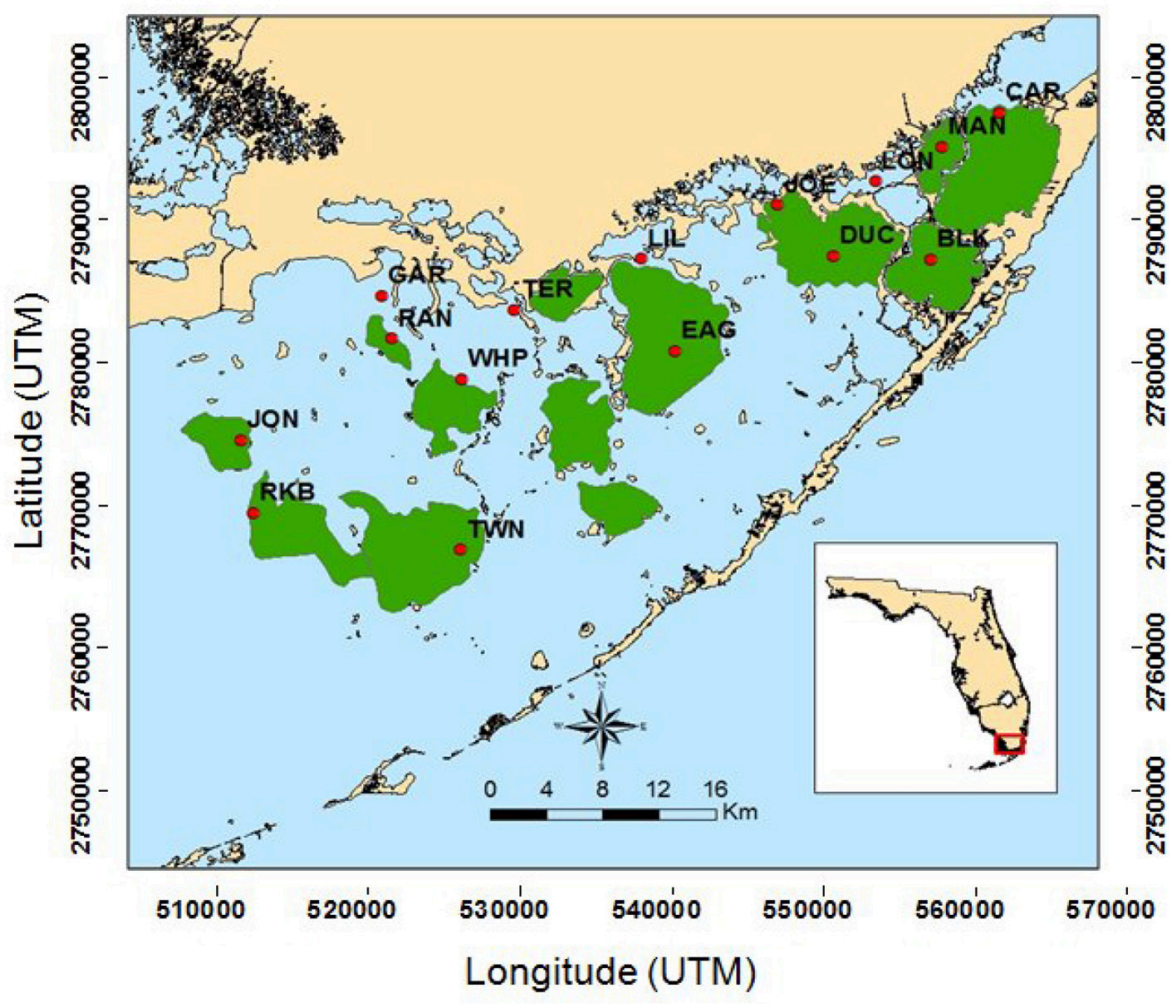
Map of South Florida Fisheries Habitat Assessment Program (FHAP-SF) sample basins (green) and the 15 permanent transect locations (red dots).

During the extremely dry summer of 2015, Florida Bay exhibited another die-off event. Garfield Bight, Johnson Key Basin, Rankin Lake, Rabbit Key Basin, and Whipray Bay (Figure 1) were affected by massive, acute seagrass die-off similar to the event that occurred in the late 1980's. This die-off occurred during a period of high temperatures and high salinities due to severe drought conditions (Hall et al., 2016). Schmidt (2002) also reported the occurrence of a “die-off” of *T. testudinum* exposing bottom muds in Rankin Lake during the drought period of 1974–1975. Thus, periodic die-offs associated with droughts and hypersalinity seem to be recurring events in this region of Florida Bay.

Here, we used multivariate analyses to examine year-to-year and basin-scale interactions between water quality and macrophytes at 15 permanent transect locations that were co-located in basins with existing long-term water sampling stations (Figure 1). Our goal was to determine which water quality variables, and their specific thresholds, best explained variation in the macrophyte communities across Florida Bay. Knowledge of which water quality and biological factors increase susceptibility to die-off can help inform CERP-related water management policies in this region.

## Materials and methods

### Sampling methods

**Figure 2:**
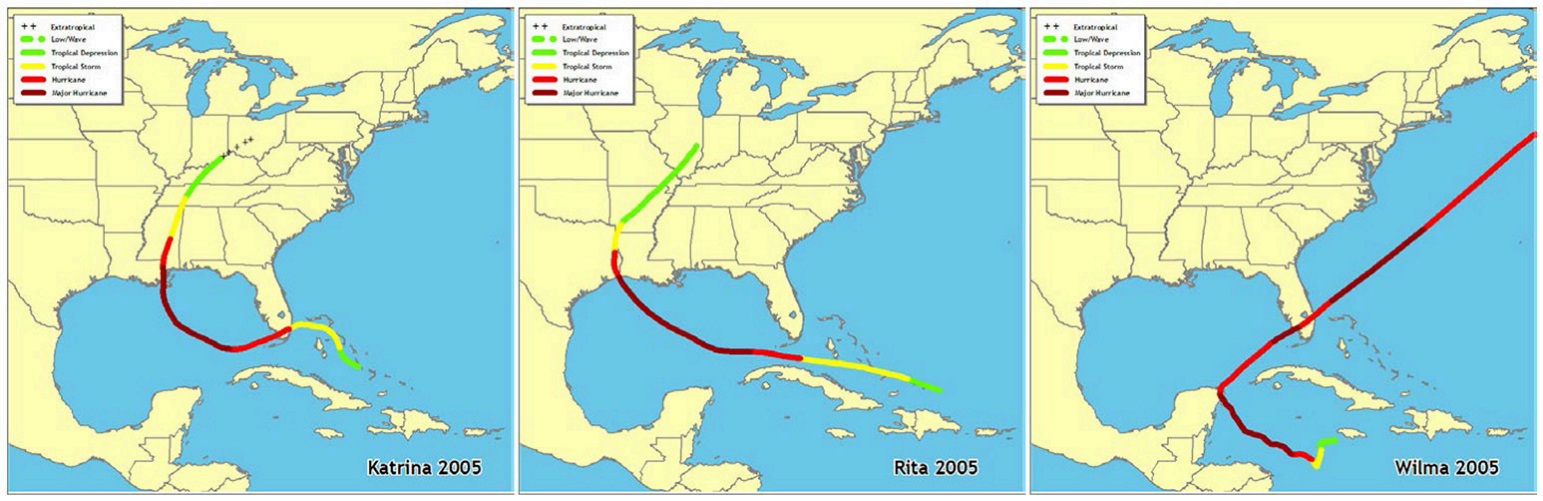
Paths of hurricanes Katrina, Rita, and Wilma that tracked near Florida Bay in fall 2005 (http://www.nhc.noaa.gov/outreach/history/).

Fifteen permanent 50 m transects were established in 2006 in Florida Bay (Figure 1, Table 1) by the Florida Fish and Wildlife Conservation Commission as part of the South Florida Fisheries Habitat Assessment Program (FHAP). Transect sampling began the year following the most active North Atlantic hurricane season ever recorded (Virmani and Weisberg, 2006) and three category 5 hurricanes (Katrina 26 August, Rita 20 September, and Wilma 24 October) passed close to Florida Bay in the fall of 2005 (Figure 2). The transect sites were co-located with pre-existing water quality sampling stations that have been sampled since 1989. Macrophyte data used in these analyses were collected at the permanent transect sites in spring (May) and fall (October) from 2006 to 2013. At each location, 0.25 m^2^ quadrats were sampled at 10 random positions along the 50 m transect. Data were collected using a modified Braun-Blanquet (BB) cover-abundance technique (Fourqurean et al., 2003). This method scored each of the macrophyte groups on a categorical scale based on abundance or cover in each quadrat. A score of 0 indicates absence of the group. A score of 0.1 indicates a single individual, 0.5 indicates a few individuals covering <5%, 1 indicates many individuals covering <5%, 2 indicates 5–25% cover, 3 is 25–50% cover, 4 is 50–75% cover, and 5 is 75–100% cover within the quadrat.

**Table 1:** Basin codes, site names and locations for the 15 permanent transect sites.

**Basins**	**Site names**	**Latitude**	**Longitude**
BLK	Blackwater Sound	25.17405	−80.42308
CAR	Card Sound	25.27355	−80.37458
DUC	Duck Key	25.17707	−80.49157
EAG	Eagle Key Basin	25.11797	−80.59972
GAR	Garfield Bight	25.15048	−80.80922
JOE	Joe Bay	25.22447	−80.53658
JON	Johnson Key Basin	25.04247	−80.91482
LIL	Little Madeira Bay	25.17517	−80.62692
LON	Long Sound	25.22737	−80.46167
MAN	Manatee Bay	25.25103	−80.41517
RAN	Rankin Lake	25.12138	−80.80288
RKB	Rabbit Key Basin	25.00242	−80.9001
TER	Terrapin Bay	25.14037	−80.71612
TWN	Twin Key Basin	24.97767	−80.75352
WHP	Whipray Bay	25.09142	−80.75478

Braun-Blanquet scores were converted to percent cover values before analysis because cover-abundance categories are not equidistant and thus cannot be properly analyzed by conventional multivariate methods (van der Maarel, 2007). First, the raw BB scores were converted to ordinal transfer values (OTV) of 1-9 using a “combined transformation,” which is a combination of a cover scale in angular transformation with a weighting based on abundance (van der Maarel, 1979). Then, the OTV were converted to percent cover values using the following equation:

$$\begin{array}{l} \ln C\;=\;\frac{\left(OTV-2\right)}{a} \end{array}$$

In this equation, *C* = cover %, OTV = 1–9 Ordinal Transfer Value, and *a* = factor weighting the cover values. The value of *a* used for this analysis is 1.380, which gives the higher Braun-Blanquet scores more weight and allows higher OTV to represent higher cover-abundance values (van der Maarel, 2007). Means of the calculated percent cover values for each transect site and sampling time were used in the multivariate analyses.

Ongoing water quality monitoring in Florida Bay has been funded by the South Florida Water Management District (SFWMD) at 28 sampling locations since 1989 (Boyer et al., 1999). Data collection was originally conducted by the Southeast Environmental Research Center (SERC) at Florida International University, and transferred to SFWMD in 2009. A subset of the larger dataset was utilized that corresponded with the 15 permanent transect sites. Details of the monthly water quality sampling and laboratory analysis have been described elsewhere (Boyer et al., 1999).

There were 36 total water variables in the original SFWMD data. The water quality variables used in this analysis were chosen based on variable inclusion criteria as follows: the variable must have been collected on at least 90% of the sampling dates (18 variables removed). Three nitrogen variables (TN, TON, TN:TP) were removed due to a change in laboratory analytical methods in 2009, which yielded significantly higher levels for TON and TN (from ≈30 to ≈50 μM 1^−1^) compared to the pre-2009 values. Water depth was also removed from the analyses because a single value was used for the first several years and actual measured depths were only recorded for the latter portion of the sampling period. For biological variables, the variable must have been collected on at least 90% of the sampling dates, and the species must have been present in 10 of the 15 sampling locations. One exception to this criterion for macrophytes is *S. filiforme*, which was present in fewer than ten sampling locations but was kept in the analysis because it is an important habitat-forming seagrass in western Florida Bay. See Tables 2, 3 for the lists of biological and water quality variables selected for this analysis.

**Table 2:** Seagrass and macroalgae variables from Braun-Blanquet cover-abundance measurements at the 15 permanent transects sites, which were used in the multivariate analysis.

TDR	Total drift red
TSG	Total Seagrass
TMA	Total Macroalgae
TT	*Thalassia testudinum*
HW	*Halodule wrightii*
SF	*Syringodium filiforme*
ACE	*Acetabularia* spp.
BAT	*Batophora* sp.
CAL	*Caulerpa* spp.
HAL	*Halimeda* spp.
PEN	*Penicillus* spp.
UDO	*Udotea* spp.
SAR	*Sargassum* spp.

**Table 3:** Water quality variables used in the multivariate analysis.

NOx-N	Nitrate + Nitrite in μm Nl^−^^1^
NH_4_-N	Ammonia in μm Nl^−1^
DIN	Dissolved inorganic nitrogen in μm Nl^−1^
${\text{PO}}_{4}^{-3}$-P	Phosphate in μm Pl^−1^
TP-P	Total phosphorus in μm Pl^−1^
CHL-A	Chlorophyll *a* in μgl^−1^
TOC	Total organic carbon in μml^−1^
SALT	Surface salinity in ppt
TEMP	Surface temperature in °C
DO	Surface dissolved oxygen in mgl^−1^
pH	pH measured in the field
TURB	Turbidity in nephelometric turbidity units
DIN:${\text{PO}}_{4}^{-3}$	Ratio of dissolved inorganic Nitrogen:Phosphate (μml^−1^)
DIN:TP	Ratio of dissolved inorganic Nitrogen:Total Phosphorus (μml^−1^)

### Statistical methods

Multivariate analyses (CLUSTER, SIMPROF, MDS, BEST, and LINKTREE) examining relationships between water quality and benthic macrophyte communities both by year and by transect location (basin) were performed using PRIMER software (version 6). The biological datasets contained data from spring 2006 through fall 2013 averaged by year, basin, and season (spring and fall). The water quality datasets contained the mean, maximum, and minimum of each variable also by year, basin, and season (6 months antecedent to the spring and fall macrophyte sampling) from December 2005 through October 2013. Logistic regressions between variations in water quality (mean, minimum and maximum) from multiple antecedent time periods (1, 3, 6, 9, and 12 months) to changes in macrophyte cover and abundance revealed that the 6-month antecedent period had the highest number of significant associations between specific water quality variables and macrophyte changes (Cole, 2017). Minimum and maximum values for water quality variables were used in addition to means to assess whether changes in the macrophyte communities were due to water quality extremes rather than just the averages (Easterling et al., 2000; Lynch et al., 2014). Water quality data were normalized so that each variable had a mean of 0 and a standard deviation of 1.

#### By-year analysis

Biological data from the permanent transects were pooled, averaged by year and then converted to a similarity matrix using a Bray-Curtis Similarity Index. This index better represents similarities or dissimilarities among communities than parametric approaches due to the lack of normality and unequal variance distribution characteristic of biological data (Clarke and Gorley, 2006). Water quality data were also pooled, averaged by year, and a similarity matrix was constructed using Euclidean distances.

Hierarchical cluster analysis was completed using the CLUSTER routine to visualize a dendrogram of similarity among years based on water quality variables. The similarity profile routine (SIMPROF) was applied to these CLUSTER analyses in order to provide stopping rules for separation of subgroups among the samples. This SIMPROF routine avoids over-interpretation of these subgroups; any groups below these stopping values are not significantly different from one another and should not be further interpreted (Clarke et al., 2008). Non-metric multi-dimensional scaling (MDS) analysis was performed to visualize similarity among basins in 2-D ordinations, based on both water quality variables and macrophyte data. MDS is considered a highly effective ordination method for community analysis (Calhoun et al., 2008). For water quality MDS analyses, similarity among years is based on mean, maximum, and minimum values pooled by year for all 14 water quality variables. For the macrophyte MDS analyses, similarity was based on the mean percent cover values of the macrophyte groups pooled by year. Due to the large separation in the MDS ordinations between 2006 and 2007 (the years following the close passage of 3 category-5 hurricanes), and the later years, additional MDS analyses were conducted with these 2 years omitted in order to better visualize the water quality and macrophyte community relationships from 2008 to 2013.

We completed the BEST non-parametric procedure as a data reduction technique to determine which water quality variables had the highest potential for explaining patterns in the biological data structures. This procedure links the two datasets by determining the water quality variables most correlated with changes in the biological communities. To minimize mutually-correlated variables, only the top 5 most highly correlated variables from the BEST procedure were selected. Using the reduced number of variables more finely resolved which abiotic variables best explained the biotic structure, without confounding effects.

CLUSTER and MDS analyses were performed again using the reduced set of water quality variables (also an additional MDS analysis using just 2008–2013), and linkage tree analysis (LINKTREE) was completed on this reduced dataset. This LINKTREE procedure uses a decision tree to identify subsets of samples from the biological resemblance matrix that are explained by thresholds in particular water quality variables within the water quality dataset. LINKTREE identified specific water quality variables and thresholds for those variables that could explain local, year-level variability in the biological communities (Calhoun et al., 2008).

Rainfall data were also examined in an attempt to determine if this environmental variable was a source of annual variation in the water quality variables or macrophyte communities. Rainfall data were obtained from the South Florida Water Management District website using the DBHYDRO tool (https://www.sfwmd.gov/science-data/dbhydro). Rainfall data in inches per day were used from the closest oceanographic stations adjacent to the existing permanent transects. These daily data were summed by year for each basin location. Basin sums were then averaged by year (± s.e.) to calculate annual bay-level rainfall averages. In total, 12 rainfall collection stations were used, as there were three cases where two of our permanent transects shared the next closest oceanographic station.

#### By-basin analysis

For the basin analysis, all of the procedures described above (except rainfall analysis) were completed, except the biological percent cover data over the study period were averaged by basin and converted to similarity matrices using Bray-Curtis Similarity Indices; the water quality data were also averaged by basin, and similarity matrices were constructed using Euclidean distances. Previous research has shown there are east-west gradients in sediment depth and total phosphorus in Florida Bay (Zieman et al., 1989; Zhang et al., 2004). Thus, among-basin relationships between sediment depth vs. total phosphorus and phosphate were examined.

## Results

### By-year analysis

**Figure 3:**
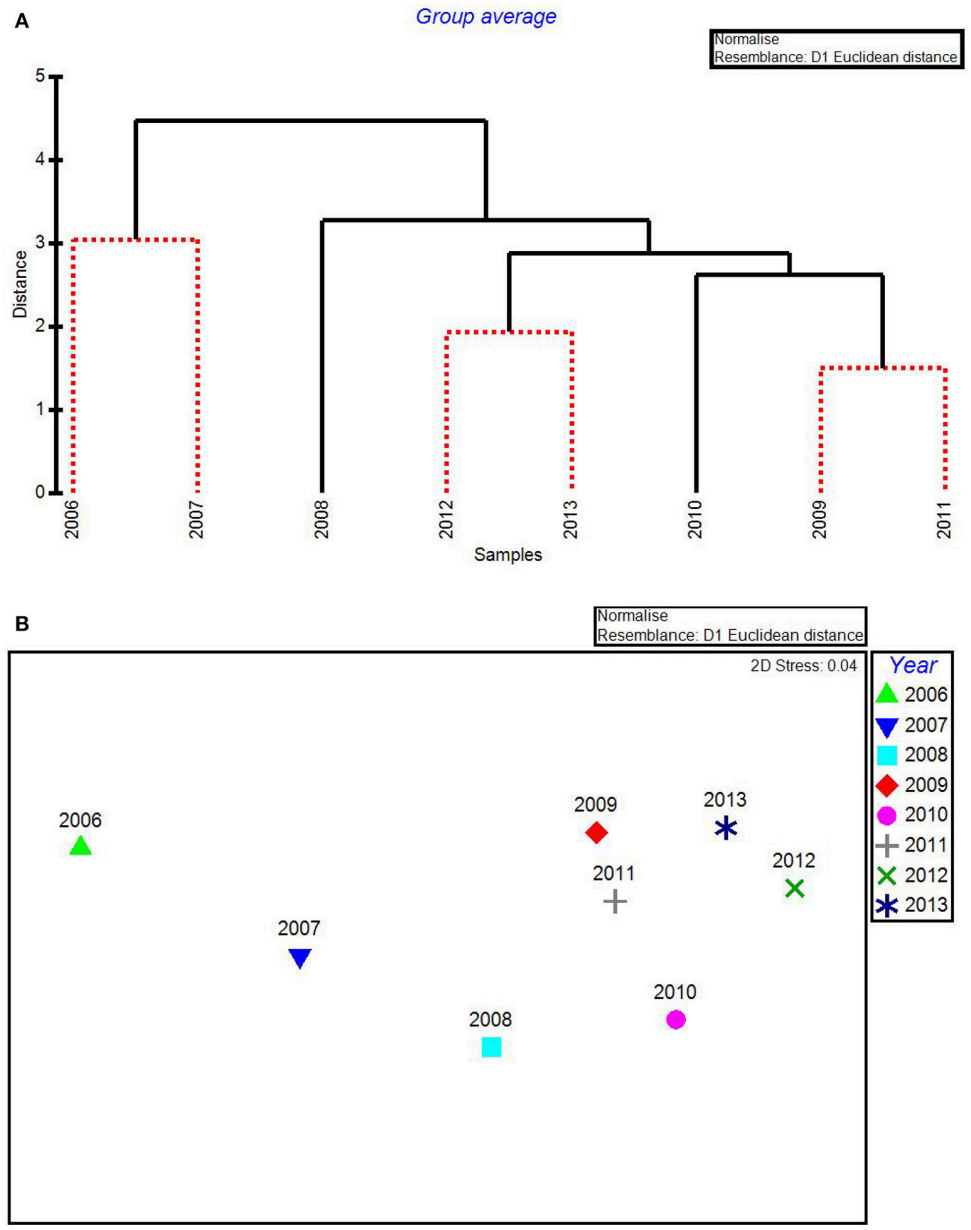
CLUSTER dendrogram **(A)** and MDS ordination **(B)** by year for all water quality variables for 2006–2013. Red lines on dendrograms indicate groups not separated by SIMPROF (*p* < 0.05).

**Figure 4:**
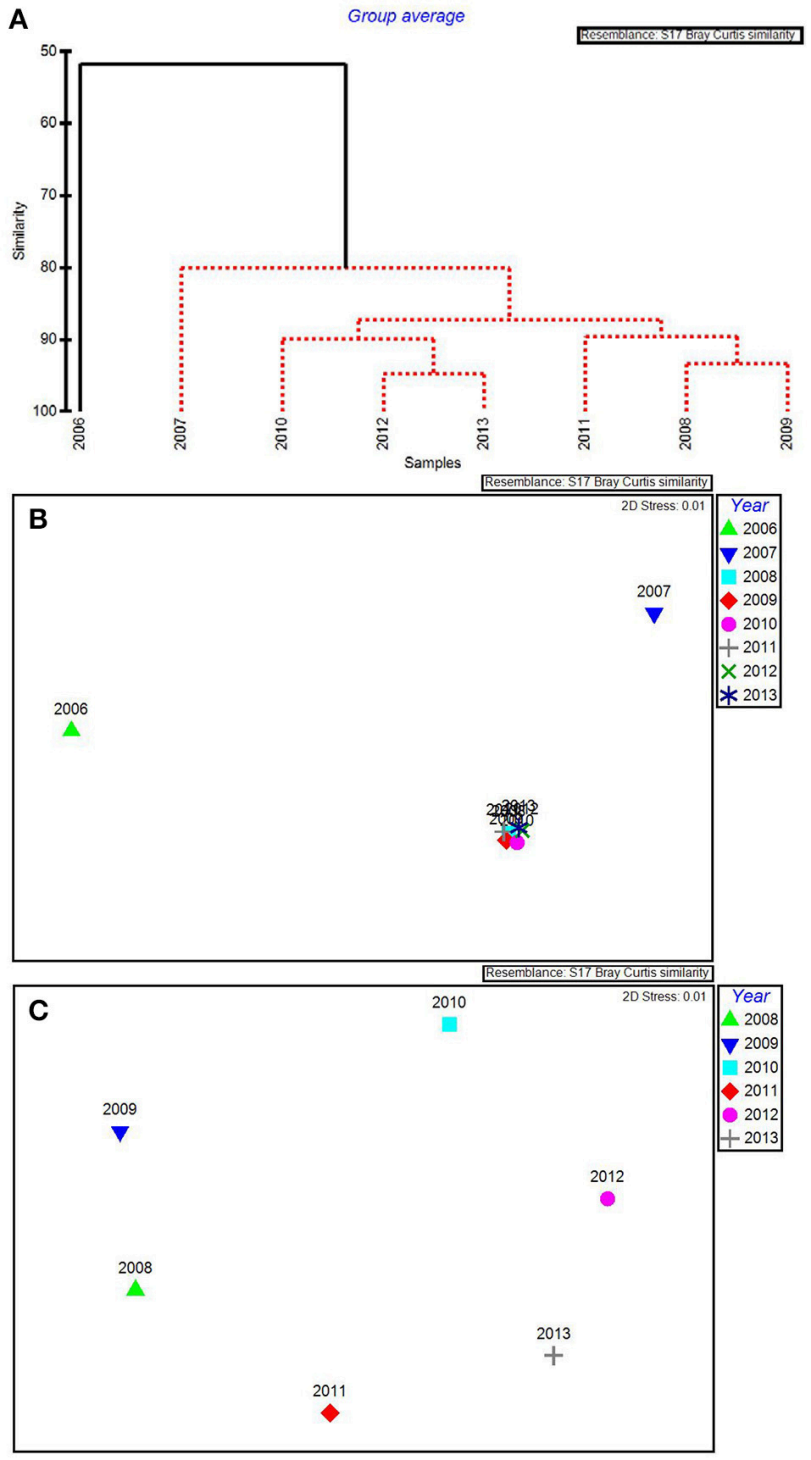
CLUSTER dendrogram **(A)** and MDS ordination **(B)** of mean percent cover values arranged by year of the macrophyte communities at 15 permanent transect sites in Florida Bay for all years 2006–2013 and an MDS ordination **(C)** with 2006 and 2007 removed. Red lines on dendrograms indicate groups not separated by SIMPROF (*p* < 0.05).

In the CLUSTER plot and MDS ordination for the environmental data (Figures 3A,B), 2006 and 2007 were separated from the later years, indicating water quality in the 2 years following the passage of the three category-5 hurricanes was most dissimilar to the subsequent 6 years. The years 2006 & 2007, 2009 & 2011, and 2012 & 2013 formed three subgroups that could not be further separated, based on SIMPROF stopping values. Macrophyte CLUSTER and MDS results also indicated that both 2006 and 2007 were highly dissimilar from the later years, suggesting the hurricanes of 2005 affected both water quality and the macrophyte communities for a 2-year period (Figures 4A,B). Due to the extreme dissimilarity between 2006 and 2007 and the later years in the study period, variation among the later years could not be detected on the MDS ordination. However, when 2006 and 2007 were excluded, 2008 to 2013 appeared to be a relatively stable period with the largest separation being between 2010 and 2011 (Figure 4C), which also formed distinct branches on the CLUSTER dendrogram (Figure 4A).

**Figure 5:**
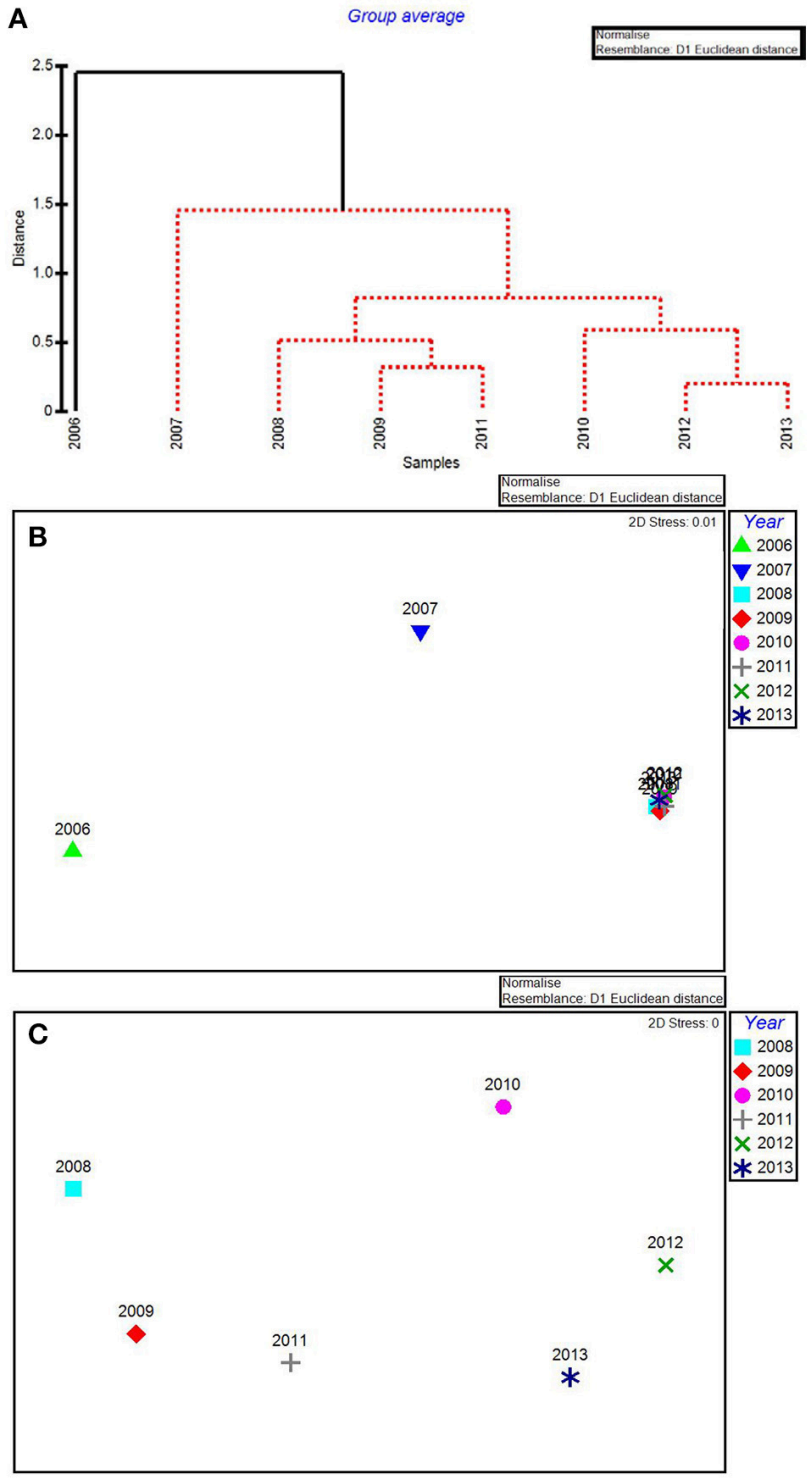
CLUSTER dendrogram **(A)** and MDS ordination **(B)** for the top 5 by-year BEST water quality variables for 2006–2013 and an MDS ordination **(C)** with 2006 and 2007 removed. Red lines on dendrograms indicate groups not separated by SIMPROF (*p* < 0.05).

The water quality variables best correlated with patterns in the biological communities were: maximum total phosphorus, mean DIN:${\text{PO}}_{4}^{-3}$, minimum chlorophyll *a*, minimum salinity, and maximum turbidity. These 5 variables were highly correlated, resulting in a ρ = 0.957 with a significance level of 0.02 (BEST analysis). Using only these water quality variables, CLUSTER analysis revealed that 2006 was still in its own subgroup, but there were no significant subgroups among the rest of the years based on SIMPROF stopping values (Figure 5A). The MDS ordination showed that both 2006 and 2007 were very dissimilar from the rest of the years (Figure 5B). The later years showed much less among-year dissimilarity (Figure 5C).

**Figure 6:**
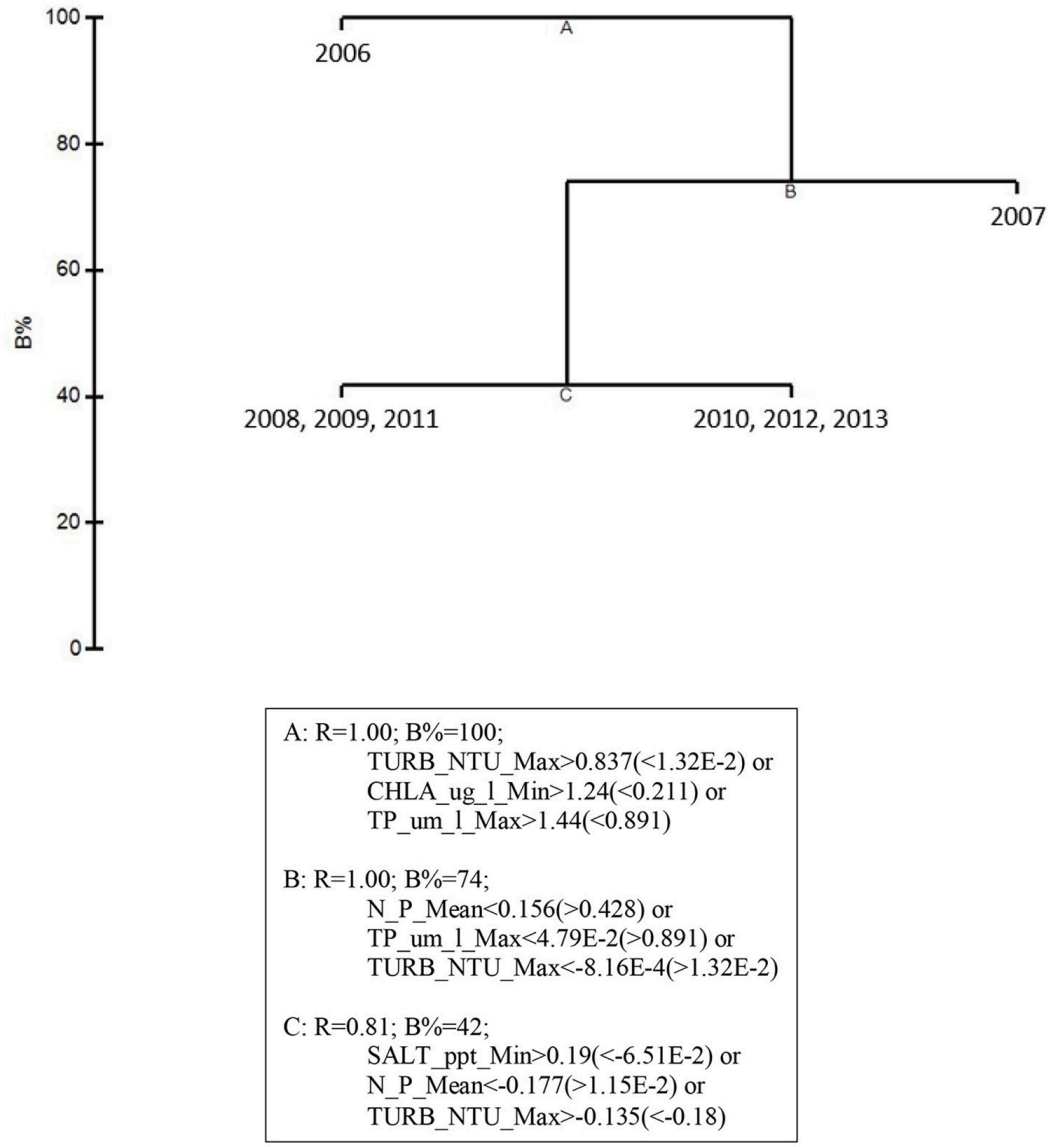
LINKTREE analysis for the top 5 by-year BEST water quality variables and macrophyte percent cover for 2006–2013 showing divisive clustering of macrophyte communities (above), constrained by inequalities in water quality variables (below).

Linkage tree analysis (LINKTREE) revealed several subsets of biological community structures among years attributable to thresholds within the 5 by-year BEST environmental variables (Figure 6). The first divisive clustering separated 2006 from the rest of the years. This division was based on thresholds in maximum turbidity (> 12.21 NTU for 2006 vs. < 6.25 NTU for the later years), minimum chlorophyll *a* (> 0.995 μg/l vs. < 0.536 μg/l), and maximum total phosphorus (TP, > 0.924 μm/l vs. < 0.740 μm/l). The second division separated 2007 from subsequent years. This division was based on mean DIN:${\text{PO}}_{4}^{-3}$ (>178.1 μm/l for 2007 vs. < 143.3 μm/l for the later years), maximum TP (>0.740 μm/l vs. < 0.457 μm/l), and maximum turbidity (>6.25 μm/l vs. < 6.15 μm/l). The final division separated 2008, 2009, and 2011 from 2010, 2012, and 2013 based on thresholds in minimum salinity, mean DIN:${\text{PO}}_{4}^{-3}$, and maximum turbidity. Minimum salinity was >28.25 for 2008, 2009, and 2011 (dry years) and < 25.95 for 2010, 2012, and 2013 (wet years); mean DIN:${\text{PO}}_{4}^{-3}$ was < 100.6 μm/l for 2008, 2009, and 2011 and >124.78 μm/l for 2010, 2012, and 2013; and maximum turbidity was >5.18 NTU for 2008, 2009, and 2011 and < 4.85 NTU for 2010, 2012, and 2013. These results suggest that both water quality and macrophyte communities in the bay were likely in an acutely disturbed phase in 2006 and were still affected in 2007, 2 years after the passage of the three category-5 hurricanes close to the bay.

#### Rainfall

**Figure 7:**
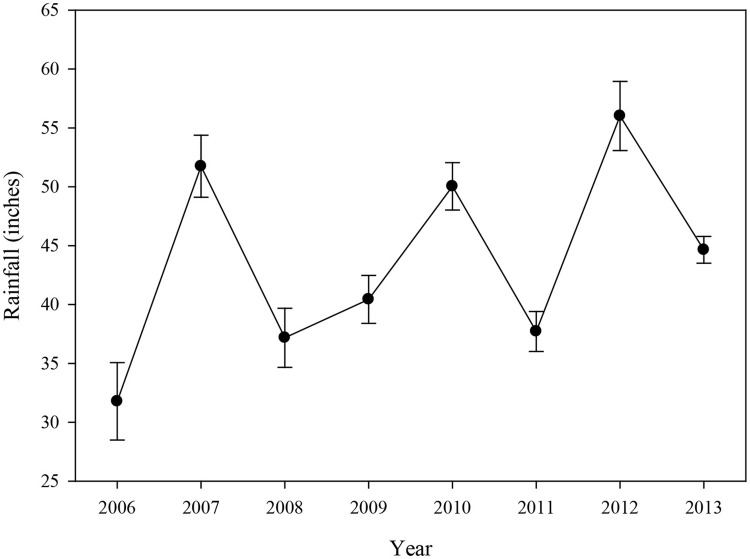
Average (±se) annual rainfall (inches) for 12 water quality stations located near the 15 permanent transects from 2006 to 2013.

Rainfall data indicated that the annual average for all basins combined was relatively high in 2007, 2010, and 2012 (Figure 7), which was primarily driven by high rainfall in Blackwater Sound, Whipray Bay, and Long Sound (data not shown). 2006, the year following the active hurricane season of 2005, had the lowest annual average rainfall during the study period.

### By-basin analysis

**Figure 8:**
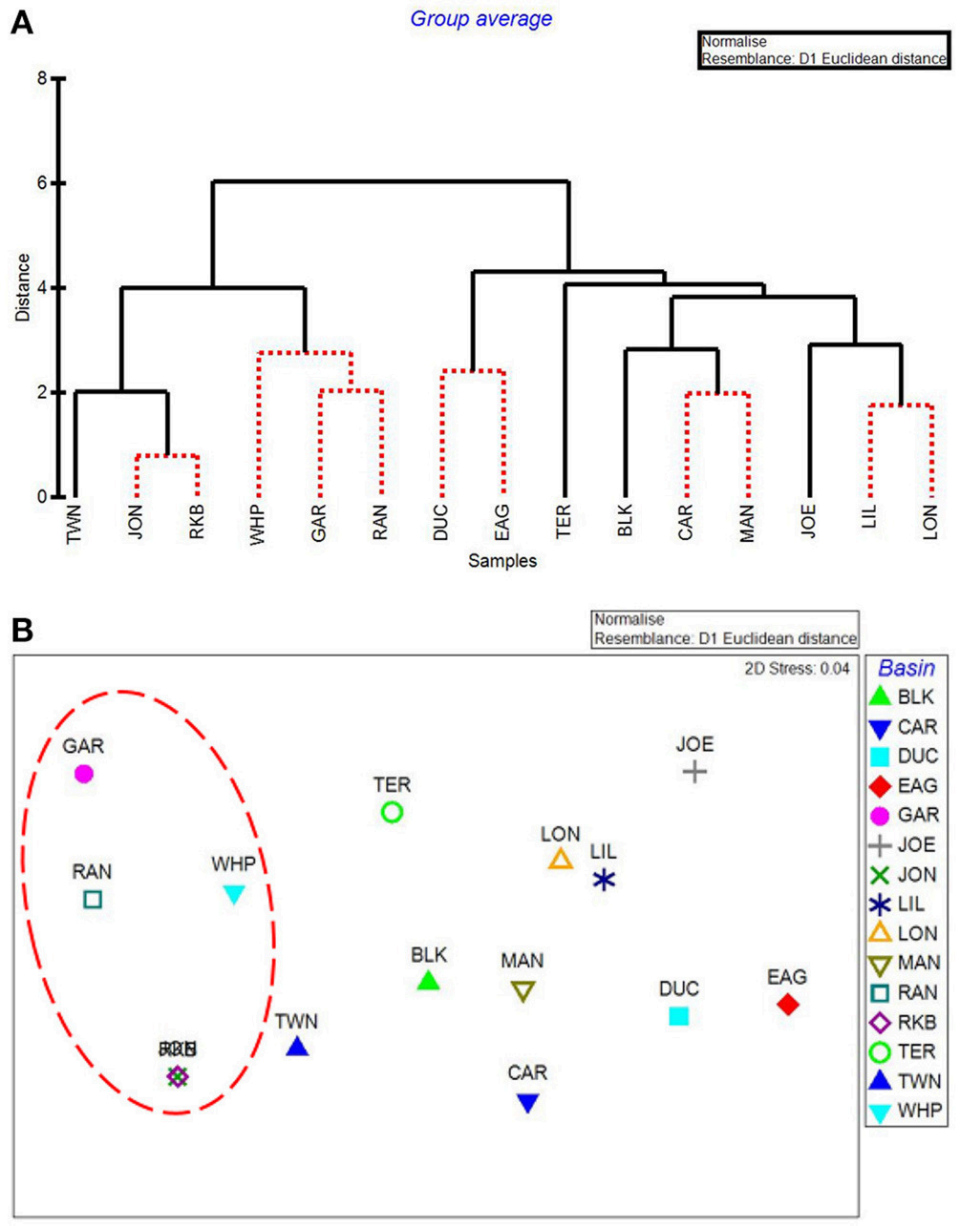
CLUSTER dendrogram **(A)** and MDS ordination **(B)** by basin for all water quality variables. Red lines on dendrograms indicate groups not separated by SIMPROF (*p* < 0.05). Red dashed line on MDS ordination surrounds basins that exhibited die-off in 2015.

CLUSTER analysis of all water quality variables analyzed by basin showed a clear split between basins in the northeast section of Florida Bay and the western/southwestern section of the bay (Figure 8A). The first major division grouped together the 5 basins that were subsequently affected by seagrass die-off in the summer of 2015 (Figure 8A, Garfield Bight, Rankin Lake, Johnson Key Basin, Rabbit Key Basin, and Whipray Bay, plus Twin Key Basin). However, Twin Key Basin was significantly different from the five die-off-affected basins. Whipray Bay, Garfield Bight, and Rankin Lake are in a separate subgroup, but they are not significantly different from one another based on SIMPROF stopping values. The other nine basins are in a second subgroup based on the first CLUSTER division. Within that large group, Terrapin Bay, Blackwater Sound, and Joe Bay are significantly different from one another and the other basins, which cannot be further separated based on SIMPROF stopping values. MDS ordination of water quality shows this same spatial trend, with the five die-off-affected basins farthest to the left on the plot (Figure 8B), with Twin Key Basin being the most similar non-die-off-affected basin.

**Figure 9:**
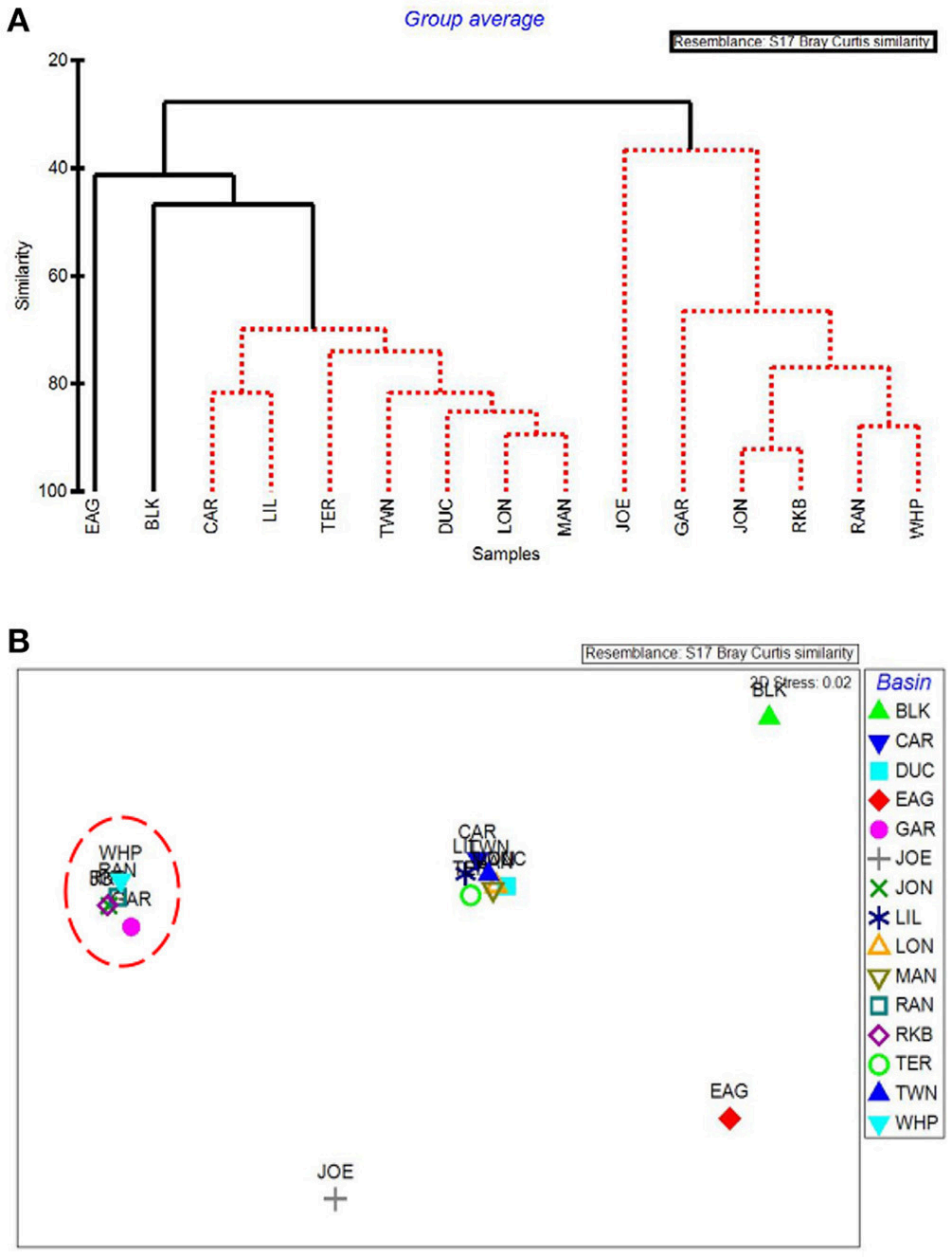
CLUSTER dendrogram **(A)** and MDS ordination **(B)** by basin for macrophyte percent cover. Red lines on dendrograms indicate groups not separated by SIMPROF (*p* < 0.05). Red dashed line on MDS ordination surrounds basins that exhibited die-off in 2015.

The first major division in the CLUSTER analysis of macrophyte cover grouped these 5 western basin communities (plus the more eastern Joe Bay) together (Figure 9A). Whipray Bay and Rankin Lake as well as Johnson Key and Rabbit Key basins are in separate subgroups, but they are not significantly different from each other based on SIMPROF stopping values. Within the second nine-basin subgroup, Eagle Key Basin and Blackwater Sound are significantly different from one another and the other seven basins, which cannot be further separated based on SIMPROF stopping values. MDS ordination of macrophyte cover also had the 5 die-off-affected basins grouping very closely to one another, indicating that these macrophyte communities were similar to one another, but dissimilar from the more eastern basins (Figure 9B).

**Figure 10:**
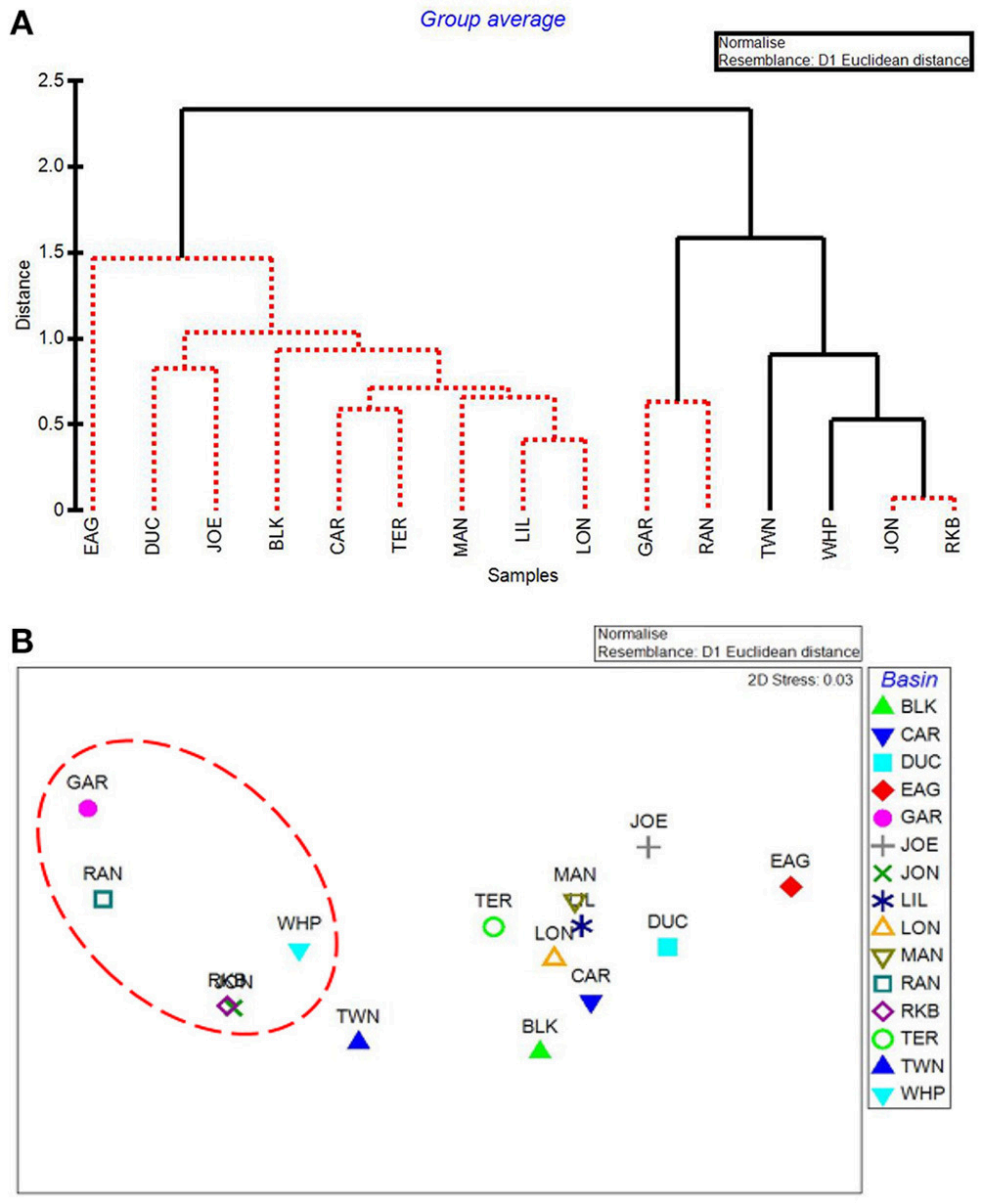
CLUSTER dendrogram **(A)** and MDS ordination **(B)** of the top 5 by-basin BEST water quality variables. Red lines on dendrograms indicate groups not separated by SIMPROF (*p* < 0.05). Red dashed line on MDS ordination surrounds basins that exhibited die-off in 2015.

The five water quality variables that best explained basin-level macrophyte cover patterns were: mean DIN, mean pH, minimum dissolved oxygen, minimum DIN:${\text{PO}}_{4}^{-3}$, and maximum dissolved oxygen, with a ρ = 0.646 and a significance level of 0.01 (BEST analysis). The first separation in CLUSTER analysis using these 5 by-basin BEST water quality variables separated Garfield Bight, Rankin Lake, Twin Key Basin, Whipray Bay, Johnson Key Basin, and Rabbit Key Basin from the other basins (Figure 10A). These basins all had die-off in summer 2015, except for Twin Key Basin. Within this group, Whipray Bay and Twin Key Basin are significantly different from one another and the other basins. Further sub-groupings were not supported based on SIMPROF. In the MDS ordination using only the 5 by-basin BEST water quality variables, die-off-affected basins were slightly more dissimilar to the other basins compared to the MDS ordination of all the water quality variables (compare Figure 8B and Figure 10B).

**Figure 11:**
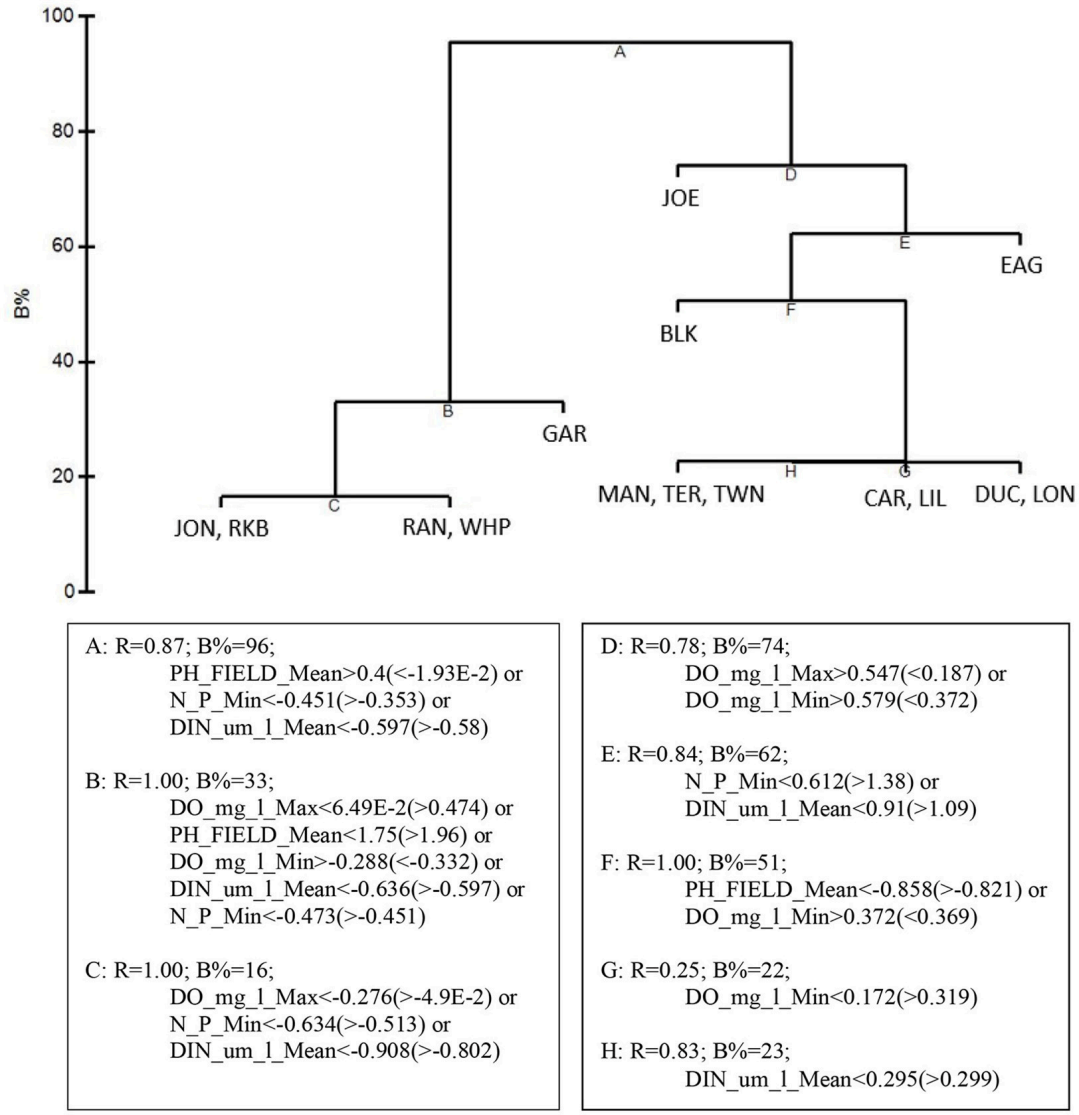
LINKTREE analysis of the top 5 by-basin BEST water quality variables and macrophyte percent cover showing divisive clustering of macrophyte communities (above), constrained by inequalities on one or more water quality variables (below).

LINKTREE analysis by basin, using the 5 by-basin BEST variables resulted in 9 divisive clusters among the 15 basins. The first separation divided all five die-off-affected basins: Garfield Bight, Johnson Key Basin, Rankin Lake, Rabbit Key Basin, and Whipray Bay, from the other 10 basins (Figure 11). This branch was determined by mean pH (>8.29 for the die-off-affected basins vs. < 8.219 for the other basins), minimum DIN:${\text{PO}}_{4}^{-3}$ (<20.08 μm/l vs. >25.07 μm/l), and mean DIN (<2.004 μm/l vs. >2.069 μm/l). The other two by-basin BEST water quality variables did not show any distinct differences between the die-off-affected basins and the other basins.

#### Sediment depth

**Figure 12:**
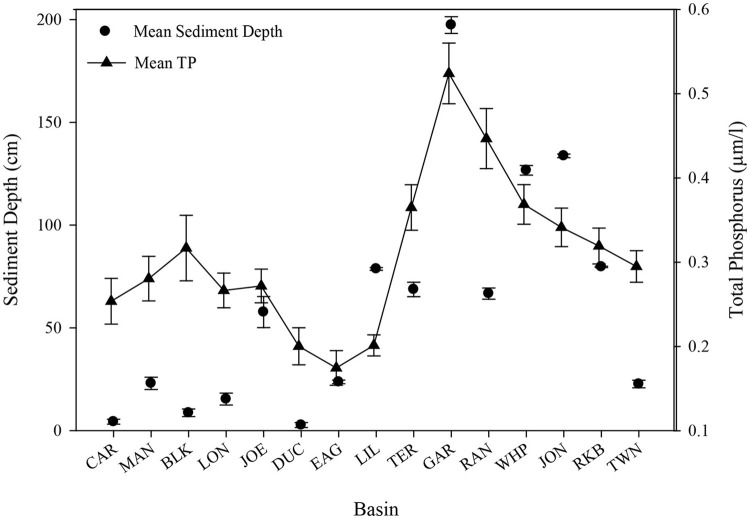
Sediment depth (cm) and mean (±se) total phosphorus (μm/l) for all 15 permanent transect locations in approximate geographic order from northeast to southwest.

Basins in Florida Bay with deeper sediments and the densest seagrasses have higher levels of phosphorus (Zieman et al., 1989; Zhang et al., 2004). Sediment depth (Figure 12) was greatest at Garfield Bight, Johnson Key Basin, and Whipray Bay, which were three of the five basins that had die-off. The other two die-off-affected basins, Rankin Lake and Rabbit Key Basin were among the top 7 basins with the greatest sediment depths. When arranged in approximate geographic order from northeast to southwest, a clear trend is evident between sediment depth and total phosphorus, with the exception of Twin Ken Basin, which has very low sediment depths (Figure 12) and did not exhibit die-off in 2015. Regression analyses indicated that total phosphorus (TP) had a significant positive relationship with sediment depth at these 15 permanent transect sites (TP = 0.237 + 0.0012^*^sediment depth, *r*^2^ = 0.51, *p* = 0.003).

### Temporal and spatial patterns

**Figure 13:**
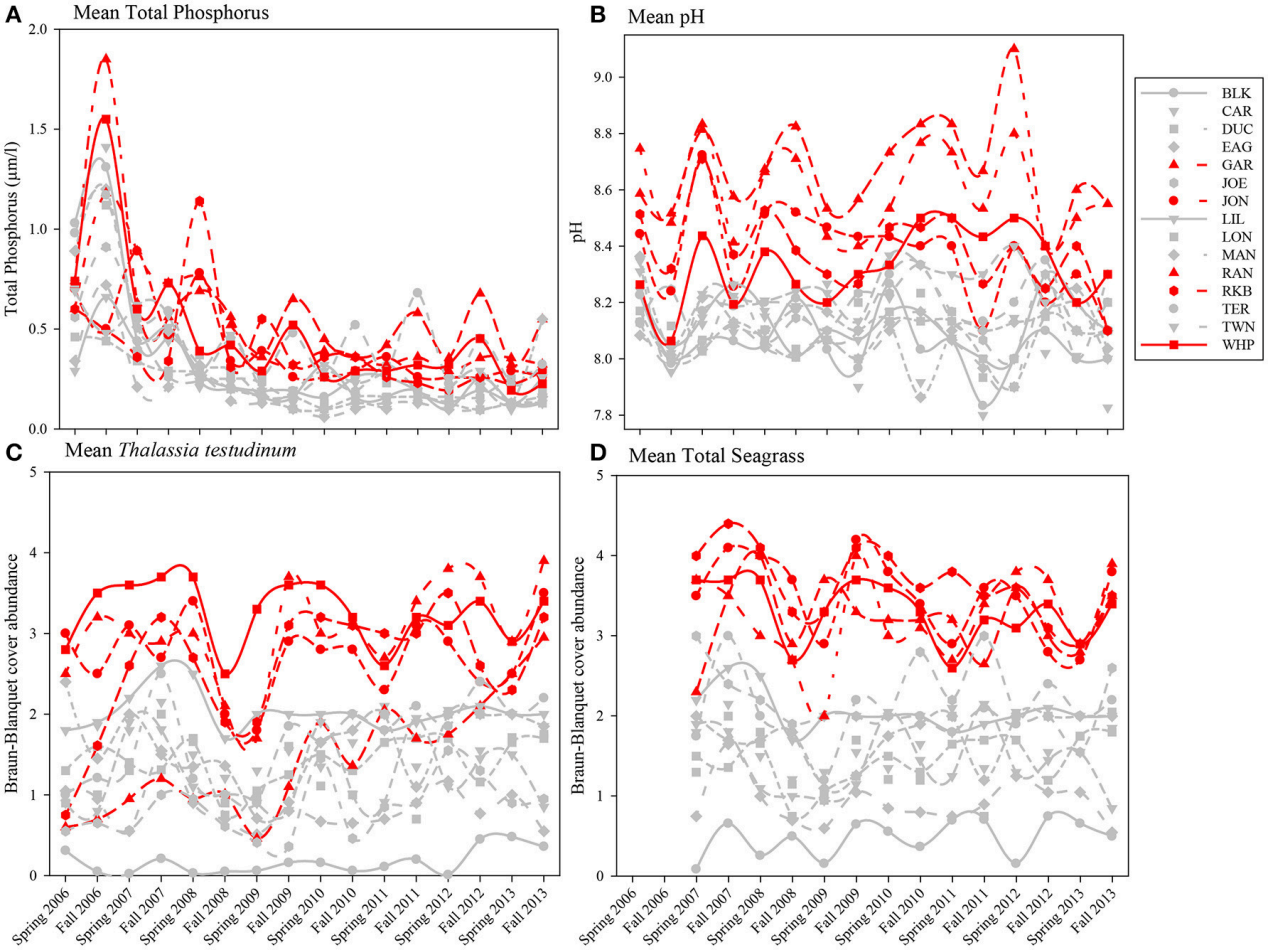
Average total phosphorus **(A)**, pH **(B)**, and Braun-Blanquet cover-abundance values for *T. testudinum* **(C)** and total seagrass **(D)** for all 15 permanent transects from 2006 to 2013. The five basins that were affected by seagrass die-off in 2015 are highlighted in red.

Because total phosphorus and pH were among the variables most affecting macrophyte communities in the multivariate analyses, seasonal means of total phosphorus, pH, *Thalassia testudinum* abundance, and total seagrass abundance were plotted by year, season, and basin. Total phosphorus levels for both spring and fall from 2006 to 2013 for individual permanent transects showed that the 5 die-off-affected basins had higher values of total phosphorus for much of the time period preceding the die-off in summer 2015 (Figure 13A). This trend was even more evident in mean pH (Figure 13B); pH was generally 0.2–0.5 higher in the 5 die-off-affected basins than the rest of the basins for the majority of the study period. The elevated pH was likely a result of high photosynthetic activity from the dense *Thalassia testudinum* (and total seagrass) at these die-off-affected locations (Figures 13C,D).

## Discussion

### By-year

Multivariate analyses of both water quality and macrophyte communities at 15 permanent transect locations across Florida Bay indicated that they were very different in 2006 and 2007 compared to 2008 through 2013. This dissimilarity is very likely due to the effects of three major category-5 hurricanes (Katrina, Rita, and Wilma) that passed near Florida Bay in the fall of 2005. Rainfall and wind associated with disturbances such as hurricanes can result in increased nutrient-rich freshwater runoff as well as nutrient and sediment resuspension in Florida Bay (Sheng et al., 1995; Hansen et al., 1997; Lawrence et al., 2004). In this regard, the highest levels of total phosphorus, chlorophyll *a*, and turbidity were observed at the permanent transect locations in 2006 relative to 2007 through 2013 (Cole, 2017). Hurricane disturbances can also affect macrophyte community species diversity due to burial and removal; the seagrass *S. filiforme* and calcareous macroalgae, such as *Penicillus* spp. and *Udotea* spp., are the most susceptible to these types of physical disturbances (Cruz-Palacios and van Tussenbroek, 2005). This type of benthic community response occurred in south Florida after the passage of Hurricane Georges in the fall of 1998 when *T. testudinum* was much less affected than *S. filiforme* and calcareous macroalgae species (Fourqurean and Rutten, 2004).

The 5 by-year BEST water quality variables explaining temporal variation in the macrophyte communities were mean DIN:${\text{PO}}_{4}^{-3}$, minimum chlorophyll *a*, minimum salinity, maximum total phosphorus, and maximum turbidity. Previous logistic regression analysis (Cole, 2017) also indicated that total phosphorus, turbidity and salinity had significant relationships with seagrasses and macroalgae in Florida Bay. Although not significant in this analysis, salinity is an important variable affecting macrophytes in Florida Bay (Fourqurean et al., 2003; Herbert and Fourqurean, 2009), and it is the primary target variable for CERP water management in the region (Rudnick et al., 2005). Florida Bay is a phosphorus-limited system, so it was not surprising that phosphorus availability, as reflected by mean DIN:${\text{PO}}_{4}^{-3}$ and maximum total phosphorus, would be important in affecting macrophyte communities (Fourqurean and Zieman, 1992; Armitage et al., 2005; Herbert and Fourqurean, 2009). In addition, the biological response variable, chlorophyll *a*, mirrors the patterns of phosphorus (as well as turbidity), due to phosphorus limitation in Florida Bay (Boyer et al., 1999). High turbidity levels are associated with low seagrass abundance in Florida Bay (Cole, 2017). The negative effect of turbidity may be due to the high light requirements of seagrasses (Duarte, 1991), but this negative effect may be mitigated in Florida Bay because sediment resuspension is a major source of phosphorus to the water column in this phosphorus-limited system (Zhang et al., 2004). Alternatively, the negative relationship between seagrass abundance and turbidity in this shallow system could reflect seagrasses acting as ecosystem engineers, reducing turbidity by slowing water movement and trapping suspended particles (Adams et al., 2016).

Linkage tree analysis (LINKTREE) helped to further characterize how the top 5 water quality variables differed in their effects on the macrophyte communities among years. Over the 8-year study period, 2006 had the highest values for maximum turbidity (>12.21 NTU), minimum chlorophyll *a* (>0.995 μg/l), and maximum total phosphorus (>0.924 μm/l). All three of these variables were likely at elevated levels due to the hurricane effects from the previous fall (Sheng et al., 1995; Hansen et al., 1997; Lawrence et al., 2004). Rudnick et al. (2006) hypothesized that elevated chlorophyll levels in eastern Florida Bay in 2006 reflected both hurricane effects and nutrient (TP and TOC) inputs associated with the widening of US Highway 1. Even by 2007, maximum total phosphorus (>0.740 μm/l), maximum turbidity (>6.25 NTU), and mean DIN:${\text{PO}}_{4}^{-3}$ (>178.1) values were still higher than the later years, reflecting long water residence times in much of Florida Bay (Nuttle et al., 2000). The extremely high DIN:${\text{PO}}_{4}^{-3}$ ratios in 2007, coupled with lower chlorophyll *a* levels (which were not different from the later years), may reflect the net uptake of dissolved phosphate by the recovering macrophyte communities causing a reduction in the high phytoplankton biomass of the previous year (i.e., following the hurricane-induced bloom). The patterns revealed by the MDS ordinations and LINKTREE analysis both indicate that the hurricane season of 2005 affected water quality and macrophyte communities in Florida Bay for approximately a 2-year period.

Increased rainfall typically affects water quality in several ways. For example, high rainfall can decrease salinity or may increase nutrient levels due to runoff from land. Rainfall over the permanent transect sites was high in 2007, which could have contributed to higher DIN:${\text{PO}}_{4}^{-3}$, total phosphorus, and turbidity levels due to mixing and runoff. Rainfall was low in 2008 and 2011, likely explaining the high average salinity during those years. Rainfall was high again in 2010 and 2012 corresponding to lower salinity during those years. However, the patterns of rainfall did not exactly match the salinity or nutrient patterns, so it appears that rainfall cannot solely be used as an explanation for year-to-year dissimilarity within these analyses. Rainfall combined with anthropogenic factors (canal discharges) have been shown to influence salinity in this region (Hunt and Nuttle, 2007).

### By-basin

Our results indicated that the water quality variables analyzed here, as well as the cover and abundance of plant communities, were distinct among basins, but that basins in close proximity to each other tended to be more similar. Individual basins in Florida Bay are physically separated from each other by mud flats and mangrove islands, and these landforms create spatial heterogeneity for organisms within the landscape (Turner et al., 2001). Landforms affect flow of water and nutrients through a system and constrain spatial patterns and processes, so certain basins may respond differently than others to an increase in freshwater due to the spatial heterogeneity and reduced connectivity among individual basins (Swanson et al., 1988). One exception to the spatial pattern was Blackwater Sound, which is located in the eastern portion of Florida Bay, but had water quality that tended to resemble more central basins. In addition, Blackwater Sound had relatively unique macrophyte communities. Hackney and Durako (2004) observed that morphometric characteristics of *T. testudinum* from Blackwater Sound also resembled those of plants from central Florida Bay rather than adjacent eastern basins. They suggested that this relatively deep basin is very diverse biologically and hydrologically, and thus resembles a microcosm of Florida Bay.

Basins in the western portion of Florida Bay are greatly influenced by the Gulf of Mexico and are the least phosphorus limited (Fourqurean and Zieman, 1992). The basins in the northeastern portion of the bay do not have this oceanic connection, but instead are more influenced by rainfall, evaporation, and freshwater inflow from the Everglades and mainland Florida (Kelble et al., 2007). CLUSTER and MDS analyses of water quality and the macrophyte communities also indicated differences between the western and eastern basins. The MDS ordination of the macrophytes closely grouped the five basins: Garfield Bight, Johnson Key Basin, Rankin Lake, Rabbit Key Basin, and Whipray Bay, which were subsequently affected by die-off in the summer of 2015 (Hall et al., 2016). This indicates that the plant communities at these basins shared similar macrophyte community structures, but were distinct from the other study basins, years before the most recent die-off occurred. The 5 water quality variables that best explained basin-level patterns in the macrophyte communities were mean DIN, mean pH, minimum dissolved oxygen (DO), minimum DIN:${\text{PO}}_{4}^{-3}$, and maximum DO. As mentioned above, Florida Bay is a phosphorus-limited system (Fourqurean and Zieman, 1992). The DIN:${\text{PO}}_{4}^{-3}$ ratio indicates the relative degree of phosphorus limitation; lower values for this ratio (and low DIN) imply that these basins had more available phosphorus, allowing a drawdown of nitrogen. The positive relationships with pH and DO indicate that seagrasses in Florida Bay may be acting as ecosystem engineers by raising pH and DO through their photosynthetic activity (Unsworth et al., 2012; Buapet et al., 2013; Hendriks et al., 2014).

Using just the top 5 water quality variables, all die-off-affected basins (plus Twin Key Basin) were again grouped together by the CLUSTER analysis (Figure 10A). Twin Key Basin did not have die-off in 2015, but it had similar water quality conditions to the other five western basins (at least based on these 5 water quality variables). This is possibly because of its western location, close proximity to the other 5 basins, and connection to the Gulf of Mexico. Twin Key Basin likely did not group with the die-off basins in the MDS ordination of the macrophyte communities (Figure 10B) because it has low sediment depths (Figure 12) and more hard bottom than those five basins, leading to more sparse *T. testudinum* and the presence of corals (Landry, 2005; Chartrand and Durako, 2009).

LINKTREE analysis helped to further characterize how the differing macrophyte communities of these five basins were associated with different thresholds in the top 5 water quality variables. Die-off-affected basins had higher mean pH (>8.29), lower minimum DIN:${\text{PO}}_{4}^{-3}$ (<20.08), and lower mean DIN (<2.004 μm/l) than the other 10 basins. The high pH in these basins likely reflects high photosynthetic activity due to dense seagrasses; the nutrient thresholds indicated they were less phosphorus-limited. Since water quality is measured monthly vs. only twice per year for macrophyte sampling, the thresholds of these three water quality variables could be used as near-real-time indicators for determining areas that may be susceptible to die-off.

Sediment depth was greatest at Garfield Bight, Whipray Bay, and Johnson Key Basin, which are three of the basins that had extensive die-off in the late 1980s and the summer of 2015. The other two die-off-affected basins, Rankin Lake and Rabbit Key Basin, were still among the seven basins with the greatest sediment depths. In Florida Bay, total seagrass abundance, sediment depth, and phosphorus availability are all highly spatially correlated (Zieman et al., 1989; Landry, 2005). Thus, deep sediments and less phosphorus limitation resulted in the highest *T. testudinum* and total seagrass cover among the 15 transects.

### Temporal and spatial patterns

The multivariate approach we used revealed how both water quality and the macrophyte communities responded to perturbations associated with the passage of three category-5 hurricanes close to Florida Bay during the extremely active hurricane season in the fall of 2005. In addition, we were able to identify specific factors that distinguished basins that subsequently exhibited die-off in 2015 from non-affected basins. Basins affected by seagrass die-off in summer 2015 displayed higher total phosphorus, higher pH, higher *T. testudinum* abundance, and higher total seagrass abundance throughout most of the study period, which ended more than 2 years before this die-off event. High seagrass abundance, primarily driven by abundance of the large-bodied *T. testudinum* in Florida Bay, may increase pH (and DO) due to photosynthetic activity. Seagrasses may thus function as ecosystem engineers by buffering ocean acidification impacts in shallow coastal areas (Unsworth et al., 2012; Buapet et al., 2013; Hendriks et al., 2014; Camp et al., 2016). Phosphorus-limited basins with lower abundance of *T. testudinum* and more mixed communities that include *H. wrightii, S. filiforme*, and more macroalgae seem to be less susceptible to die-off during high temperature and high salinity events, which is consistent with the CERP goals of promoting more heterogeneous communities (Rudnick et al., 2005).

In conclusion, the combined factors of deep sediments, high total phosphorus, high pH, low DIN:${\text{PO}}_{4}^{-3}$, and abundant total seagrass cover, dominated by *T. testudinum*, appear to lead to increased susceptibility to seagrass mortality during late-summer drought conditions when temperatures and salinities are high. Although restoration actions related to increasing freshwater inflow to Florida Bay have been initiated by CERP, water management changes thus far have not led to significant decreases in salinity in portions of the bay; salinities in Rankin Lake, Johnson Key Basin and Whipray Bay reached 15 year highs immediately preceding the most recent seagrass die-off (Hall et al., 2016). Without further efforts to release fresh water into these at-risk basins during drought periods, die-off events may continue to recur in these western Florida Bay basins.

## Author contributions

AC, MD, and MH together designed and executed the research. MH supervised the field sampling. AC and MD conducted the statistical analyses and drafted the manuscript with the assistance of MH. All co-authors commented on and approved the final manuscript draft.

### Conflict of interest statement

The authors declare that the research was conducted in the absence of any commercial or financial relationships that could be construed as a potential conflict of interest.
